# An Analysis of the Role of Bisphenol A in Breast and Reproductive-System Cancers

**DOI:** 10.3390/jcm14134706

**Published:** 2025-07-03

**Authors:** Maria Derkaczew, Kamila Zglejc-Waszak, Lukasz Dabrowski, Janusz Kocik, Adam Zdaniukiewicz, Michael Thoene, Marcin Jozwik, Slawomir Gonkowski, Joanna Wojtkiewicz

**Affiliations:** 1 Students’ Scientific Club of Pathophysiologists, Department of Human Physiology and Pathophysiology, School of Medicine, University of Warmia and Mazury in Olsztyn, 10-082 Olsztyn, Poland; 2 Department of Anatomy and Histology, School of Medicine, Collegium Medicum, University of Warmia and Mazury in Olsztyn, 10-082 Olsztyn, Poland; kamila.zglejc@uwm.edu.pl; 3 Clinical Hospital of Ministry of Interior with Warmia-Mazury Cancer Center, 10-228 Olsztyn, Poland; lukaszdabrowski.com@gmail.com (L.D.); jkocik@cmkp.edu.pl (J.K.); ord.chir.piersi@poliklinika.net (A.Z.); 4 School of Public Health, Center of Medical Postgraduate Education, 00-416 Warsaw, Poland; 5 Department of Medical Biology, Faculty of Health Sciences, University of Warmia and Mazury in Olsztyn, 10-561 Olsztyn, Poland; michael.thoene@uwm.edu.pl; 6 Department of Gynecology and Obstetrics, School of Medicine, Collegium Medicum, University of Warmia and Mazury, 10-045 Olsztyn, Poland; marcin.jozwik@uwm.edu.pl; 7 Department of Clinical Physiology, Faculty of Veterinary Medicine, University of Warmia and Mazury in Olsztyn, Oczapowskiego 13, 10-718 Olsztyn, Poland; slawomir.gonkowski@uwm.edu.pl; 8 Department of Human Physiology and Pathophysiology, School of Medicine, Collegium Medicum, University of Warmia and Mazury in Olsztyn, 10-082 Olsztyn, Poland

**Keywords:** bisphenol A, breast cancer, reproductive system cancer

## Abstract

**Background/Objectives**: Bisphenol A (BPA) is an organic compound used in producing polycarbonates and epoxy resins found in products such as food containers, disposable bottles, CDs, and DVDs. Its structure resembles that of endogenous estrogen, which classifies BPA as an endocrine-disrupting chemical (EDC). BPA has been associated with various health abnormalities, including cancer and reproductive system cancer. In this study, we examine the association between BPA exposure, BPA levels in blood serum, and the occurrence of breast cancer and reproductive system cancer. **Methods**: A total of 84 females were included in this cross-sectional study. All participants completed a questionnaire assessing BPA exposure and underwent a blood test to measure BPA levels in serum. **Results**: Analysis of the lifestyle questionnaire revealed behavioral differences potentially associated with BPA exposure. A statistically significant difference was observed for responses to Question 13, related to food preparation methods, while responses to Questions 5, 6, and 17 showed trends approaching statistical significance in cancer groups. Serum BPA concentrations were significantly higher in patients with reproductive system cancer compared to the control group (*p* = 0.045), while a non-significant trend was observed between breast cancer patients and patients with reproductive system cancer (*p* = 0.0884). **Conclusions**: In summary, our study demonstrated significantly elevated serum BPA levels in patients with reproductive system cancer compared to controls. These results suggest the hypothesis that higher exposure to BPA may influence or be associated with the development of estrogen-dependent cancers such as breast and endometrial cancer. However, due to the cross-sectional design of the study, causality cannot be established, and further longitudinal studies are warranted.

## Simple Summary

Bisphenol A is a chemical commonly used in plastic products such as food containers, bottles, and packaging. Because its structure is similar to that of natural estrogen, it may interfere with hormonal balance in the human body. Some researchers believe that long-term exposure to BPA may increase the risk of developing certain types of cancer, especially those related to hormones. In this study, we examined women with breast cancer and cancer of the reproductive system to see if they had higher levels of BPA in their blood serum. We also asked participants about daily habits that might affect exposure to this chemical. Our results showed that women with cancer had higher levels of BPA compared to healthy individuals; in addition, certain behaviors relating to activities such as food storage and preparation were more common in these groups. These findings suggest a possible link between lifestyle habits, BPA exposure, and hormone-related cancers.

## 1. Introduction

Bisphenol A (BPA, 2,2-Bis(4-hydroxyphenyl) propane) is an organic compound from the phenol group which is used to produce plastics. BPA is utilized in the manufacture of polycarbonates (PCs) and epoxy resins [1]. PCs are a group of polymers used to produce items such as food contact materials, disposable bottles, plastic containers, can coatings, CDs, DVDs, electronic housings, and household appliances [2,3]. The main source of human exposure to BPA is food, specifically through migration of this compound from packaging into food products. The main sources of BPA are plastics used for packaging of food and beverage products and for the lining of cans [4]. Experiments have shown that BPA in canned products accumulates mainly in solid portions of food rather than in liquids [5]. Household dust has been found to contain bisphenol A, suggesting that exposure to this compound may also occur via inhalation of intoxicated air and dust [6,7].

Moreover, BPA’s structure closely resembles that of estrogen to the extent that it is often referred to as synthetic estrogen. Long-term observation of the effects of BPA has made it one of the most frequently studied endocrine-disrupting chemicals (EDCs) [8,9]. Xenoestrogens, which are among the EDCs, are substances of natural or synthetic origin that harm the endocrine system by disrupting the production of endogenous hormones and the normal functioning of hormonal axes [10]. BPA is compatible with nuclear estrogen receptors (ERα and Erβ) and also binds to membrane estrogen receptors (mERs) and G protein-coupled receptors (GPR30 and GPER). Additionally, it exhibits antagonistic activity toward thyroid hormone (thyroxin) receptors as well as a moderate anti-androgenic potential [9]. Disruption of the body’s hormonal balance leads to various abnormalities, and BPA’s ability to mimic estrogens contributes to the development of estrogen-dependent cancers such as breast cancer, endometrial cancer type 1, and ovarian cancer [11]. It has been shown that fetal exposure to BPA in mice can significantly impact the development of neoplastic lesions, including changes in the mammary gland, potentially increasing susceptibility to breast cancer later in life [12].

Breast cancer is one of the most widespread malignant tumors affecting women, and is the primary cause of death among women of reproductive age. In the female population, the leading locations for cancers remain the breast, colon, lungs, and uterus. In the Polish statistics for 2023, lung cancer continued to be the leading cause of cancer-related deaths in women (18%), ahead of breast cancer (16%). However, when looking at incidence rates, breast cancer ranked first (23%), with lung cancer in second place (9%), uterine cancer in third (7%), and ovarian cancer in sixth (4%). Poland is among the countries whose population faces a high risk of breast cancer, with an increasing trend over time having also being reported [13,14,15]. The occurrence of breast cancer is associated with unhealthy lifestyle habits, environmental influences, and socio-psychological factors. The majority of risk factors for cancer development are modifiable. Estrogens, both endogenous and exogenous, play a significant role in breast cancer development. While the ovaries are the primary source of estrogen, postmenopausal fat tissue also produces small amounts, with higher levels of body fat increasing estrogen exposure and breast cancer risk. Hormone replacement therapy (HRT) and oral contraceptives further contribute to estrogen-related risk, with studies showing elevated breast cancer incidence among users, though the risk decreases after discontinuation [16]. Reproductive system cancers, such as endometrial cancer type 1 and ovarian cancer, are estrogen-dependent. Due to BPA’s ability to mimic estrogen and act as a so-called “endocrine disruptor”, it is suggested that BPA may contribute to the development of these cancer types as well. It is also suggested that BPA may influence the development of type 1 endometrial cancer through indirect stimulation of abnormal proliferation of endometrial cells [17]. The latest studies highlight the potential role of BPA in affecting the cellular and non-cellular components surrounding the tumor which form the so-called tumor microenvironment (TME) which strongly affects tumor progression and metastasis [11].

BPA has been detected in various human and animal tissues and bodily fluids, including blood serum, urine, saliva, and the mammary gland. However, exposure to BPA among residents of the Warmian–Masurian voivodeship in Poland has not yet been studied. Therefore, this project aimed to assess BPA levels in the blood serum of clinically healthy patients and compare them with the levels found in patients suffering from breast and reproductive system cancer. Furthermore, we aimed to compare the results of a survey assessing each patient’s exposure to BPA with incidence rates for the aforementioned cancers.

## 2. Materials and Methods

### 2.1. Study Setting and Patients

We performed a cross-sectional study to determine the association between BPA exposure, BPA blood plasma level, and the occurrence of breast and reproductive system cancers (endometrial and ovarian cancers). The study was conducted in the Clinical Hospital of the Ministry of Internal Affairs and Administration with the Warmia-Mazury Oncology Centre in Olsztyn (departments of Gynecologic Oncology and Urogynecology, Breast Subunit), as well as the Oncology and Immuno-oncology Department with the Day Oncology Therapy Unit in the years 2022–2024. A group of patients treated in the aforementioned departments underwent a survey and an examination of BPA levels in blood serum.

All participants provided their informed consent before their inclusion in the study. The study included a structured 23-item questionnaire developed by the authors to assess dietary habits, food packaging preferences, and environmental exposure to plastic-related materials (Table 1).

Each affirmative answer scored one point. The questions covered various aspects of lifestyle potentially related to BPA exposure, including frequency of processed food intake, use of plastic containers, consumption of canned food, drinking habits involving plastic or metal packaging, cleaning habits, and having a CD/DVD collection. We conducted face-to-face interviews with patients to ensure that each survey was completed correctly. Patients’ weights and heights were measured, and BMI was calculated. Vital signs were checked including blood pressure. In addition to the survey-based assessment, blood samples were collected from all patients.

### 2.2. Blood Sample Collection

Blood samples were collected from all study participants using standard venipuncture techniques. Blood was drawn into sterile, additive-free Vacutainer tubes to ensure sample integrity. Immediately after collection, the tubes were kept at room temperature for 30 min to allow clot formation.

Following coagulation, the blood samples were centrifuged at 3000 rpm for 10 min at 4 °C to separate the serum. The obtained serum was then carefully aliquoted into sterile microtubes and stored at −80 °C until further ELISA testing. This procedure ensured the stability of BPA levels for subsequent quantification.

### 2.3. Enzyme-Linked Immunosorbent Assay

The measurement of Estrogen BPA Environmental in plasma was measured using a commercially available Estrogen BPA Environmental ELISA Kit (Abcam, ab175820, Cambridge, UK). The procedure was carried out strictly according to the manufacturer’s protocol. Samples and standards were plated in duplicate. The calculations were made according to the determined curve and formula.

### 2.4. Statistical Analysis

Statistical analysis was performed in GraphPad Prism version 8 and also in MedCalc version 23.1.3. A *p*-value of less than 0.05 was considered statistically significant. The analysis of the distribution allowed the selection of the appropriate test. Analyses were performed using one-way ANOVA with Tukey’s post hoc test, Student’s *t*-test, or nonparametric equivalents.

## 3. Results

### 3.1. Patients’ Characteristics

**Figure 1:**
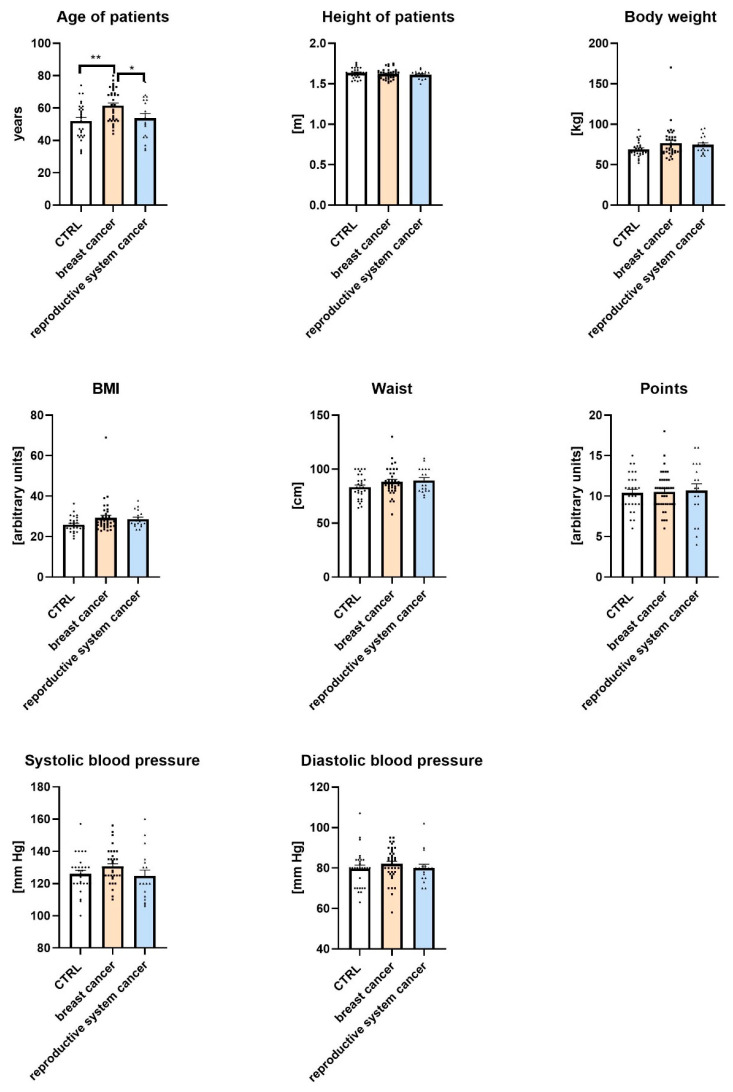
Demographic and anthropometric characteristics of control (CTRL), breast cancer, and reproductive system cancer groups. Data are expressed as means  ±  SEM; * *p* ≤ 0.05, ** *p* ≤ 0.01 (** = 0.0025, * = 0.0417). Values of *n* per group are as follows: *n* = 29 in CTRL (control group); *n* = 37 in breast cancer group; *n* = 18 in reproductive system cancer group. *n* values are indicated by dots.

The study included 84 female patients, classified into three groups: control (CTRL; *n* = 29), breast cancer (*n* = 37), and reproductive system cancer (*n* = 18). All participants were women. Ages ranged from 32 to 80 years, with a mean age of 56.55 years. Patients’ demographic and anthropometric data divided into CTRL, breast cancer, and reproductive system cancer groups are presented in Figure 1.

As shown in the figure, there were statistically significant differences in the ages of participants across groups. Women with breast cancer were significantly older than women in the CTRL group and women in the reproductive system cancer group (*p* < 0.01 and *p* < 0.05, respectively). No significant differences were observed among the groups in terms of height, body weight, BMI, waist circumference, or health-related points. Similarly, systolic and diastolic blood pressure values did not differ significantly between the studied groups.

### 3.2. Questionnaire-Based Analysis of Patient Lifestyle and Environmental Exposure

Lifestyle and environmental exposure were assessed using a 23-question survey. The frequency of affirmative responses (scored as 1 point each) was compared across patient groups: CTRL, breast cancer, and reproductive system cancer.

The results of the questionnaire-based analysis are summarized in Table 2, which compares the frequencies of affirmative responses to each question given by individuals in the CTRL group, the breast cancer group, and the reproductive system cancer group. Odds ratios (ORs), 95% confidence intervals (CIs), and *p*-values were calculated using the CTRL group as the reference category.

Among the analyzed parameters, no statistically significant differences were observed in the prevalence of self-reported hypertension between groups. Hypertension was reported by 37.93% of women in the CTRL group, 56.76% in the breast cancer group, and 38.89% in the reproductive system cancer group. Although the prevalence appeared higher among patients with breast cancer, this difference did not reach statistical significance (OR = 1.0413, 95% CI: 0.3110–3.4871, *p* = 0.1313).

Among the questionnaire items, the majority of responses did not significantly differ between groups. However, patients diagnosed with breast cancer were significantly less likely to answer affirmatively to Question 13, which addressed the use of specific food preparation methods (e.g., frying or grilling), compared to the CTRL group (24.32% vs. 48.28%; OR = 0.3444, 95% CI: 0.1210–0.9804, *p* = 0.0458). This finding may suggest differences in dietary habits that warrant further investigation. In addition, a borderline trend toward statistical significance was observed for Question 5, which concerned the frequency of eating in fast-food restaurants. Patients with reproductive system cancer were more likely to report this practice compared to controls (27.78% vs. 6.9%; OR = 5.1923, 95% CI: 0.8859–30.4317, *p* = 0.0679), indicating a potential link to increased exposure to endocrine-disrupting compounds. Similarly, affirmative responses to Question 6, regarding buying fruits and vegetables in plastic bags, showed a lower frequency among breast cancer patients compared to controls (8.11% vs. 24.14%, OR = 0.2773, 95% CI: 0.0647–1.1881, *p* = 0.0840), while affirmative responses to Question 17, asking whether water from a plastic bottle accounted for more than 1 L of daily fluid intake, showed a higher frequency in the breast cancer group than in controls (51.35% vs. 27.59%, OR = 2.7708, 95% CI: 0.9807–7.8285, *p* = 0.0544).

Differences between groups in their responses to certain questions exhibited trends which approached statistical significance (Questions 5, 6, 13, and 17). Several additional trends were also observed. For example, to Question 16, which addressed not paying attention to the chemical composition of food storage containers, women with reproductive system cancer were less likely to respond affirmatively compared to the CTRL group (33.3% vs. 58.62%, OR = 0.3529, 95% CI: 0.1034–1.2044, *p* = 0.0963), indicating a possible behavioral difference that warrants further investigation. No other questionnaire items revealed statistically or near-statistically significant differences between groups. While some odds ratios appeared elevated, particularly for low-frequency responses (e.g., Question 12, OR = 5.0571), the associated confidence intervals were wide and *p*-values non-significant, limiting the interpretability of these results. Overall, the findings suggested that a few specific behaviors related to food preparation and storage may have differed between cancer and CTRL groups, while most lifestyle factors assessed in the questionnaire appeared similarly distributed.

### 3.3. Categorical Comparisons Using the Chi-Square Test

To further assess group-level differences in behavioral patterns, a chi-square test (χ^2^) was performed for each questionnaire item, comparing the distribution of affirmative (“yes”) versus negative (“no”) responses among the CTRL group, breast cancer patients, and reproductive system cancer patients.

Statistically significant differences in response distributions were observed for several questions. Notably, responses to Question 3, which assessed processed food intake frequency, revealed a highly significant difference between the breast cancer and reproductive system cancer groups (χ^2^ = 38.30, *p* < 0.0001), indicating a marked disparity in reported behaviors between these two populations. Responses to Question 5, regarding the frequency of eating in fast-food restaurants, showed a significant difference between the same two groups (χ^2^ = 7.69, *p* = 0.0055). A significant difference was also found in responses to Question 13, related to food preparation methods (e.g., frying or grilling), between the CTRL and breast cancer groups (χ^2^ = 4.05, *p* = 0.0443), reinforcing earlier findings from odds ratio analysis. Finally, responses to Question 17, which referred to water from a plastic bottle accounting for more than 1 L of the daily fluid intake, differed significantly between breast cancer and reproductive system cancer groups (χ^2^ = 4.15, *p* = 0.0417), potentially indicating variation in exposure to BPA-containing linings.

In addition to these significant findings, several other items (e.g., Questions 6 and 16) demonstrated *p*-values approaching significance, suggesting further possible behavioral trends warranting further study. The full results of the chi-square tests are presented in Table 3.

These results, consistent with previous findings obtained using logistic regression, support the presence of behavioral differences between groups in specific areas related to food storage and preparation which may be linked to BPA exposure. While some significant differences were also observed between the two cancer groups, these findings are reported for descriptive purposes only, and are not emphasized in the Discussion or Conclusions, as the primary focus of the study was on differences relative to the healthy CTRL group.

### 3.4. Serum BPA Concentration

**Figure 2:**
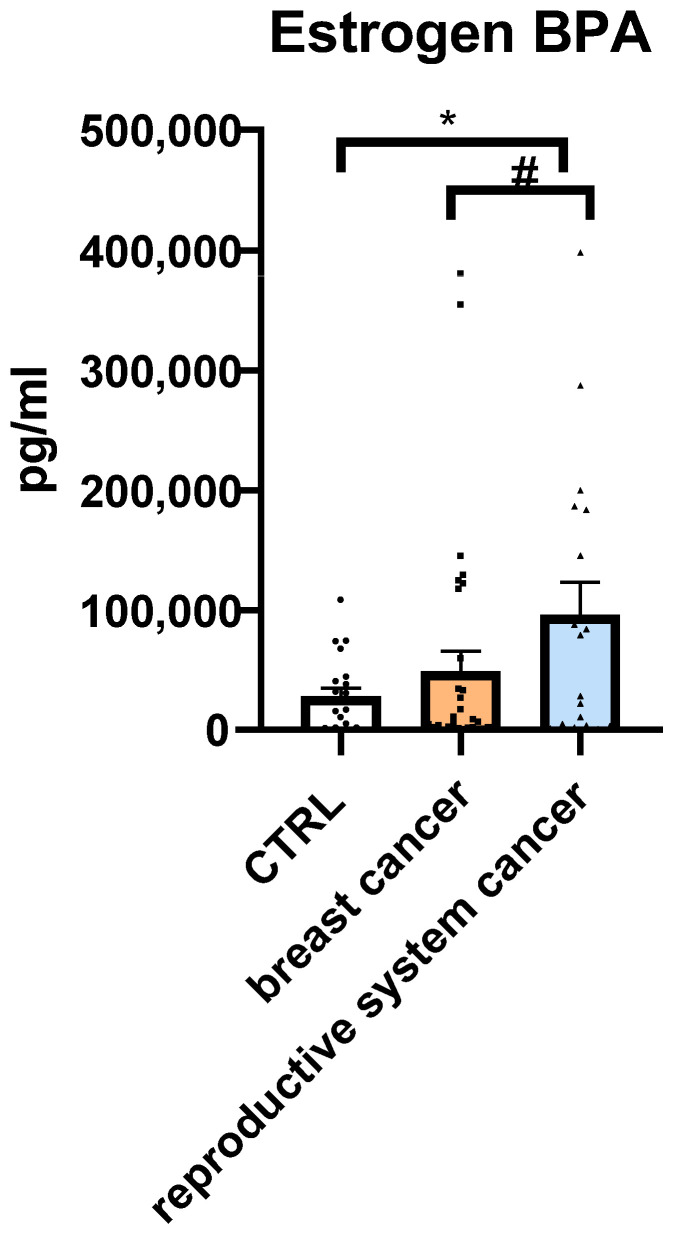
Serum Bisphenol A (BPA) concentrations [pg/mL] in the control group (CTRL), breast cancer group, and reproductive system cancer group. Median values are indicated. Differences were analyzed using the Mann–Whitney U test. Data are expressed as means  ±  SEM; * *p*  ≤  0.05 (* = 0.045; # = 0.0884). Values of *n* per group: *n* = 29 in CTRL (control group), *n* = 37 in breast cancer group, *n* = 18 in reproductive system cancer group. *n* values are indicated by dots.

Serum levels of BPA were measured in all participants using ELISA. Due to the non-normal distribution of the data, as confirmed by the Shapiro–Wilk test (*p* < 0.05 for all groups), non-parametric Mann–Whitney U tests were applied to compare BPA concentrations between groups (Figure 2; Table 4).

Mean BPA concentration was lowest in the CTRL group (28,191.2 pg/mL), second-lowest in the breast cancer group (49,136 pg/mL), and highest in the reproductive system cancer group (95,883.4 pg/mL).

A statistically significant difference in serum BPA levels was found between patients with reproductive system cancer and the CTRL group (*p* = 0.045), with the former exhibiting a more-than-threefold increase in median BPA concentration. Although patients with breast cancer showed a nearly twofold higher mean BPA level than CTRL, this difference did not reach statistical significance (*p* = 0.877). The comparison between reproductive system cancer and breast cancer groups also approached significance (*p* = 0.0884), suggesting a possible trend toward higher BPA exposure in the reproductive cancer group.

These findings support a potential association between elevated serum BPA levels and the presence of hormone-sensitive cancers, particularly in patients with reproductive system malignancies

## 4. Discussion

This cross-sectional study investigated the relationship between blood serum BPA concentrations and the presence of hormone-sensitive cancers in women. The findings showed that patients with reproductive system cancer exhibited significantly elevated serum BPA levels compared to healthy controls. In addition, those with breast cancer exhibited a nearly twofold increase in BPA levels; however, this result was not statistically significant. These observations align with prior evidence linking BPA, a well-known EDC, to estrogen-dependent malignancies such as estrogen receptor-positive breast cancer, endometrial cancer type 1, and selected subtypes of ovarian cancer, such as low-grade serous and endometrioid tumors [11,18,19,20,21,22]. Behavioral differences captured through the lifestyle questionnaire further support this association. Notably, the increased use of plastic containers and canned foods among cancer patients, particularly those with reproductive cancers, points to possible environmental sources of BPA exposure. Overall, while most differences between the study groups were not statistically significant, selected trends in dietary behavior and food storage practices may suggest behavioral risk patterns associated with BPA exposure. These preliminary findings support the need for larger, longitudinal studies to explore these associations further.

Our findings align with previous research results indicating that dietary intake is the primary source of BPA exposure in the general population [10,23,24]. Although the questionnaire did not isolate exposure categories by source type, variables related to dietary habits—including heating food in plastic (Q13), storing it in plastic packaging (Q14), and consuming bottled drinks (Q17)—were most strongly associated with elevated serum BPA levels based on odds ratios and chi-square tests. These results suggest that dietary intake might be the predominant contributor to systemic BPA burden in this population.

Our study assessed BPA levels directly in blood serum using ELISA. This approach allows for the detection of circulating, biologically active BPA. Our findings revealed significantly elevated serum BPA concentrations in patients with reproductive system cancer compared to healthy controls. Nevertheless, our results showed a trend toward lower levels of BPA in serum harvested from breast cancer patients compared to patients with reproductive system cancer. These results support the hypothesis that internal BPA burden may be linked to the development of hormone-dependent malignancies, potentially through endocrine-disrupting mechanisms. Our findings are consistent with those of previous studies, including a recent investigation conducted in Bangladesh, which demonstrated that serum BPA levels in breast cancer patients were four to seven times higher than in healthy controls, with average values ranging from 17.0 to 34.0 ng/mL compared to 4.0 ng/mL in the CTRL group. That study also reported a statistically significant association between elevated BPA concentrations in blood serum and the severity of cancer-related symptoms [13]. In another study, BPA was detected in urine and breast adipose tissue samples from the studied populations. The higher average BPA concentration in cancer patients compared to non-cancerous individuals suggests that BPA may contribute to an increased risk of breast cancer [25]. This highlights the need for public health initiatives aimed at reducing plastic use and emphasizes the importance of conducting larger studies in diverse population groups [13].

Focusing on the endocrine-disrupting potential of BPA, numerous studies have highlighted its ability to mimic endogenous estrogens. BPA disrupts the hormonal system primarily by interacting with estrogen receptors, leading to dysregulation of estrogen signaling pathways. Beyond its association with cancer, BPA has been shown to induce early onset of puberty, affect reproductive systems in both females and males, and contribute to increased body weight. Mechanistically, BPA interacts with estrogen-related receptors (ERRs), binds to cell membrane-associated receptors, modulates the expression of cytochrome P450 genes, alters estradiol levels, influences DNA methylation patterns, and activates peroxisome proliferator-activated receptors (PPARs). These multifaceted molecular interactions may contribute to its broad range of physiological and pathological effects [26,27,28]. Exposure to BPA has been associated with the development of several types of cancer, especially those originating in hormone-sensitive tissues, including those of the breast, prostate, testes, ovaries, and endometrium. Early-life BPA exposure in males may disrupt cell growth and migration, increasing the risk of testicular and prostate cancers. BPA has been shown to promote the proliferation and migration of various cell types, including those from the lung, colon, and liver. BPA exposure may activate signaling pathways such as PI3K/Akt and JAK-STAT, and disrupt normal p53 expression, particularly in breast cancer. In addition to its direct effects on tumor cells, BPA can also influence the tumor microenvironment by impairing immune surveillance, potentially accelerating breast cancer progression [29,30]. BPA can bind to classical estrogen receptors (ERα and ERβ), as well as non-classical receptors such as the G-protein-coupled estrogen receptor (GPER), mimicking endogenous estrogens and modulating downstream signaling pathways. This interaction may lead to increased cellular proliferation, inhibition of apoptosis, and altered differentiation in estrogen-sensitive tissues. Additionally, BPA exposure has been linked to epigenetic alterations, including DNA methylation and histone modifications, which can promote oncogenic gene expression patterns [27,31,32]. These observations are supported by several preclinical studies that demonstrate the role of BPA in estrogen receptor activation, epigenetic regulation, and tumor progression [33,34,35,36].

The study is limited by its sample size, particularly in the reproductive system cancer subgroup. In addition, its cross-sectional design prevents causative conclusions from being drawn. Although consistent trends are highlighted in the study results, as the questionnaire relied on self-reported data, the possibility cannot be excluded that certain behaviors were underreported or overreported, influencing the observed associations. Nevertheless, these findings underscore the need for public health initiatives focused on reducing BPA exposure, especially among at-risk populations, and encourage further prospective studies to establish causality.

## 5. Limitations

This study has several limitations. First, there was no age matching between the cancer and CTRL groups, which may have confounded patterns of BPA concentration and lifestyle exposure. The significant age difference between the CTRL and cancer groups also represents a potential confounding factor because BPA exposure and cancer risk may both increase with age. This limitation should be taken into account when interpreting the observed associations. Second, the study’s cross-sectional design limited the ability to infer causality between BPA levels and cancer incidence. Third, the relatively small sample size in the reproductive cancer group (*n* = 18) reduced statistical power and our ability to detect moderate statistical differences. The triple-negative breast cancer patients (*n* = 6) may have presented a different cancer biology to that of hormone-positive breast cancer patients. Even though triple-negative breast cancer is not hormone-sensitive, BPA has been shown to cause inflammation and increase ROS formation, contributing to p53 mutation and/or oncogene formation [37]. Because of this, and because of the low number of triple-negative breast cancer patients in our study, we decided to analyze and describe all breast cancer patients together as a single group, rather than subjecting triple-negative breast cancer patients to an independent statistical analysis. Fourth, because behaviors were self-reported, potential reporting biases were introduced. Fifth, the population sample was geographically limited to Poland, potentially affecting generalizability to broader populations. Lastly, no multivariate adjustment was made for potential confounders such as genetic predisposition or other environmental exposures. Despite these limitations, the findings offer preliminary insights into potential associations between BPA exposure and hormone-related cancers, warranting further longitudinal studies.

## 6. Conclusions

Our study measured blood serum levels of BPA in patients with breast cancer and reproductive system cancer, revealing notably elevated concentrations in both groups compared to healthy controls. These findings support the hypothesis that BPA may contribute to the development of estrogen-dependent cancers, such as breast, endometrial, and certain ovarian cancers. While not all results reached statistical significance, several trends regarding plastic-related habits suggest meaningful behavioral differences between patient groups that may have influenced BPA exposure. However, interpreting exposure patterns based solely on self-reported survey data remains challenging due to confounding factors such as age differences, lifestyle variability, and generational shifts in product use. Younger individuals may have greater exposure to plastics and electronics, yet cancer diagnoses more commonly occur later in life, potentially obscuring clear associations. Importantly, due to the cross-sectional design of the study, causality cannot be inferred. These preliminary results underscore the need for further investigation using larger and age-balanced cohorts. Longitudinal studies in which biomonitoring is integrated with detailed lifestyle assessment will be essential to clarify the role of BPA in the etiology of hormone-related cancers and inform effective public health strategies.

## Figures and Tables

**Table 1:** List of questions included in the questionnaire used in the study. Each affirmative answer scored one point. Questions covered topics related to dietary habits, food packaging, and BPA exposure risk factors.

Question Number	Description
1	A history of obesity in the family.
2	Low-calorie diet, calorie deficit currently.
3	Frequency of processed food intake.
4	Type of processed food (instant soups, instant puddings and kissels, ready-to-eat microwaveable meals, frozen pizzas, casseroles, and dumplings packed in plastic bags)
5	Frequency of eating in fast-food restaurants.
6	Buying fruits and vegetables in plastic bags.
7	Purchasing rice or groats in a cardboard package containing multiple portions in plastic bags, or inside a plastic pouch.
8	Type of fish usually consumed.
9	Regular intake of yogurts and desserts packaged in plastic containers.
10	Regular intake of plastic-bottled milk.
11	Regular intake of cheese packaged in plastic containers.
12	Consumption of canned food.
13	Method of preparing dishes (frying, grilling).
14	Storing food in plastic containers.
15	Drinks most frequently consumed during the day: mineral water in plastic bottles, soft drinks in bottles, soft drinks or coffee in cans, juices in plastic bottles.
16	Not paying attention to the chemical composition of food storage containers.
17	Water from a plastic bottle accounting for more than 1 L of daily fluid intake.
18	When purchasing drinks in plastic bottles or cans, drinking them directly from the container.
19	Frequency of consuming beer in cans (several times a week or more).
20	Having a collection of CDs/DVDs consisting of 20 or more copies.
21	Vacuuming with an electric vacuum cleaner (several times a week or more).
22	Consuming food in restaurants (several times a week or more).
23	Ordering a meal-box diet (during working days or on a daily basis).

**Table 2:** Distribution of patients among study groups and percentages of affirmative responses to individual questionnaire items. Odds ratios (ORs), 95% confidence intervals (CIs), and *p*-values are shown for comparisons with the control group. Control (CTRL). Reference—reference for OR calculation.

	Total Number of Patients					
Patient groups	CTRL 29/84 (34.52%)		Breast cancer 37/84 (44.05%)		Reproductive system cancer 18/84 (21.43%)	
Parameter/question	Number of patients: *n*, (%), OR, 95% CI, Z, *p*-value					
Hypertension	11/29 (37.93%)	reference	21/37 (56.76%)	OR: 1.0413, 95% CI: 0.3110–3.4871, Z: 1.509 *p* = 0.1313	7/18 (38.89%)	OR: 1.0413, 95% CI: 0.3110–3.4871, Z: 0.066 *p* = 0.9476
1	18/29 (62.07%)	reference	25/37 (67.58%)	OR: 1.2731, 95% CI: 0.46–3.5239, Z: 0.465 *p* = 0.642	10/18 (55.56%)	OR: 0.7639, 95% CI: 0.2313–2.5225, Z: 0.442 *p* = 0.6586
2	25/29 (86.2%)	reference	35/37 (94.6%)	OR: 2.8, 95% CI: 0.4754–16.4929, Z: 1.138 *p* = 0.2551	15/18 (83.33%)	OR: 0.8, 95% CI: 0.1570–4.0753, Z: 0.269 *p* = 0.7882
3	3/29 (10.35%)	reference	3/37 (8.11%)	OR: 0.7647, 95% CI: 0.1425–4.1024, Z: 0.313 *p* = 0.7543	1/18 (5.56%)	OR: 0.5098, 95% CI: 0.0489–5.3153, Z: 0.563 *p* = 0.5732
4	23/29 (79.31%)	reference	23/37 (62.16%)	OR: 0.4286, 95% CI: 0.1402–1.3102, Z: 1.486 *p* = 0.1372	15/18 (83.33%)	OR: 1.3043, 95% CI: 0.2822–6.0297, Z: 0.340 *p* = 0.7337
5	2/29 (6.9%)	reference	1/37 (2.7%)	OR: 0.3750, 95% CI: 0.0323–4.3535, Z: 0.784 *p* = 0.4330	5/18 (27.78%)	OR: 5.1923, 95% CI: 0.8859–30.4317, Z: 1.826 *p* = 0.0679
6	7/29 (24.14%)	reference	3/37 (8.11%)	OR: 0.2773, 95% CI: 0.0647–1.1881, Z: 1.728 *p* = 0.0840	3/18 (16.67%)	OR: 0.6286, 95% CI: 0.1398–2.8265, Z: 0.605 *p* = 0.5450
7	22/29 (75.86%)	reference	23/37 (62.16%)	OR: 0.5227, 95% CI: 0.1776–1.5382, Z: 1.178 *p* = 0.2388	13/18 (72.2%)	OR: 0.8273, 95% CI: 0.2173–3.1495, Z: 0.278 *p* = 0.781
8	24/29 (82.76%)	reference	34/37 (91.9%)	OR: 2.3611, 95% CI: 0.5145–10.8364, Z: 1.105 *p* = 0.2691	15/18 (83.33%)	OR: 1.0417, 95% CI: 0.2167–5.0071, Z: 0.051 *p* = 0.9594
9	29/29 (100%)	reference	37/37 (100%)	OR: 1.2712, 95% CI: 0.0245–65.9908, Z: 0.119 *p* = 0.9052	18/18 (100%)	OR: 0.6271, 95% CI: 0.0119–32.9913, Z: 0.231 *p* = 0.8175
10	9/29 (31.03%)	reference	15/37 (40.54%)	OR: 1.5152, 95% CI: 0.5439–4.2209, Z: 0.795 *p* = 0.4267	8/18 (44.4%)	OR: 1.7778, 95% CI: 0.526–6.009, Z: 0.926 *p* = 0.3545
11	13/29 (44.83%)	reference	18/37 (48.65%)	OR: 1.1660, 95% CI: 0.4397–3.0922, Z: 0.309 *p* = 0.7576	11/18 (61.1%)	OR: 1.9341, 95% CI: 0.5841–6.4043, Z: 0.309 *p* = 0.7576
12	0/29 (0%)	reference	2/37 (5.41%)	OR: 4.1549, 95% CI: 0.1918–89.9925, Z: 0.908 *p* = 0.364	1/18 (5.55%)	OR: 5.0571, 95% CI: 0.1951–131.0588, Z: 0.976 *p* = 0.3291
13	14/29 (48.28%)	reference	9/37 (24.32%)	OR: 0.3444, 95% CI: 0.1210–0.9804, Z: 1.997 *p* = 0.0458	9/18 (50%)	OR: 1.0714, 95% CI: 0.3304–3.4747, Z: 0.115 *p* = 0.9085
14	11/29 (37.93%)	reference	12/37 (32.43%)	OR: 0.7855, 95% CI: 0.2838–2.1740, Z: 0.465 *p* = 0.642	9/18 (50%)	OR: 1.6364, 95% CI: 0.4978–5.3794, Z: 0.811 *p* = 0.4173
15	25/29 (86.21%)	reference	32/37 (86.49%)	OR: 1.024, 95% CI: 0.2487–4.2156, Z: 0.033 *p* = 0.9738	16/18 (88.9%)	OR: 1.28, 95% CI: 0.2095–7.8189, Z: 0.267 *p* = 0.7892
16	17/29 (58.62%)	reference	19/37 (51.35%)	OR: 0.7451, 95% CI: 0.2795–1.9866, Z: 0.588 *p* = 0.5565	6/18 (33.3%)	OR: 0.3529, 95% CI: 0.1034–1.2044, Z: 1.663 *p* = 0.0963
17	8/29 (27.59%)	reference	19/37 (51.35%)	OR: 2.7708, 95% CI: 0. 0.9807–7.8285, Z: 1.923 *p* = 0.0544	4/18 (22.2%)	OR: 0.75, 95% CI: 0.1891–2.9742, Z: 0.409 *p* = 0.6823
18	10/29 (34.48%)	reference	16/37 (43.24%)	OR: 1.4476, 95% CI: 0.5301–3.9535, Z: 0.722 *p* = 0.4705	8/18 (44.4%)	OR: 1.52, 95% CI: 0.4558–5.0691, Z: 0.681 *p* = 0.4956
19	0/29 (0%)	reference	0/37 (0%)	OR: 0.7867, 95% CI: 0.0152–40.8381, Z: 0.119 *p* = 0.8175	0/18 (0%)	OR: 1.5946, 95% CI: 0.0303–83.8879, Z: 0.231 *p* = 0.9052
20	10/29 (34.48%)	reference	12/37 (32.43%)	OR: 1.2091, 95% CI: 0.3575–4.0887, Z: 0.305 *p* = 0.8608	7/18 (38.89%)	OR: 0.912, 95% CI: 0.3257–2.5536, Z: 0.175 *p* = 0.76
21	20/29 (69%)	reference	23/37 (62.16%)	OR: 0.7393, 95% CI: 0.2640–2.0702, Z: 0.575 *p* = 0.5653	10/18 (55.56%)	OR: 0.5625, 95% CI: 0.1664–1.9013, Z: 0.926 *p* = 0.3545
22	0/29 (0%)	reference	0/37 (0%)	OR: 0.7867, 95% CI: 0.0152–40.8381, Z: 0.119 *p* = 0.8175	1/18 (5.56%)	OR: 0.5619, 95% CI: 0.0217–14.5621 Z: 0.347 *p* = 0.7285
23	1/29 (3.45%)	reference	0/37 (0%)	OR: 0.2533, 95% CI: 0.0099–6.4525, Z: 0.831 *p* = 0.4058	0/18 (0%)	OR: 0.5135, 95% CI: 0.0198–13.2931, Z: 0.401 *p* = 0.6881

**Table 3:** Chi-square test (χ^2^) results comparing the distribution of questionnaire responses across study groups.

Parameter/Question	Χ^2^ CTRL vs. Breast Cancer	Χ^2^ CTRL vs. Reproductive System Cancer	Χ^2^ Breast Cancer vs. Reproductive System Cancer
Hypertension	Χ^2^ = 2.2719 DF = 1 *p* = 0.1317	Χ^2^ = 0.0042 DF = 1 *p* = 0.9482	Χ^2^ = 1.5188 DF = 1 *p* = 0.2178
1	Χ^2^ = 0.2132 DF = 1 *p* = 0.6443	Χ^2^ = 0.2132 DF = 1 *p* = 0.6443	Χ^2^ = 0.7413 DF = 1 *p* = 0.3892
2	Χ^2^ = 1.363 DF = 1 *p* = 0.243	Χ^2^ = 0.0708 DF = 1 *p* = 0.7902	Χ^2^ = 1.8243 DF = 1 *p* = 0.1768
3	Χ^2^ = 0.0969 DF = 1 *p* = 0.7556	Χ^2^ = 0.3202 DF = 1 *p* = 0.5715	Χ^2^ = 38.2963 DF = 1 *p* < 0.0001
4	Χ^2^ = 2.2293 DF = 1 *p* = 0.1354	Χ^2^ = 0.1136 DF = 1 *p* = 0.736	Χ^2^ = 2.4953 DF = 1 *p* = 0.1142
5	Χ^2^ = 0.6490 DF = 1 *p* = 0.4205	Χ^2^ = 0.6490 DF = 1 *p* = 0.0531	Χ^2^ = 7.6914 DF = 1 *p* = 0.0055
6	Χ^2^ = 3.2002 DF = 1 *p* = 0.0736	Χ^2^ = 0.3623 DF = 1 *p* = 0.5473	Χ^2^ = 0.8960 DF = 1 *p* = 0.3438
7	Χ^2^ = 1.3852 DF = 1 *p* = 0.2392	Χ^2^ = 0.0757 DF = 1 *p* = 0.7832	Χ^2^ = 0.5321 DF = 1 *p* = 0.4657
8	Χ^2^ = 1.3852 DF = 1 *p* = 0.2392	Χ^2^ = 0.3623 DF = 1 *p* = 0.5473	Χ^2^ = 0.896 DF = 1 *p* = 0.3438
9	-	-	-
10	Χ^2^ = 0.6252 DF = 1 *p* = 0.4291	Χ^2^ = 0.8467 DF = 1 *p* = 0.3575	Χ^2^ = 0.0745 DF = 1 *p* = 0.7849
11	Χ^2^ = 0.0939 DF = 1 *p* = 0.7593	Χ^2^ = 1.1534 DF = 1 *p* = 0.2828	Χ^2^ = 0.7408 DF = 1 *p* = 0.3894
12	Χ^2^ = 1.5921 DF = 1 *p* = 0.2070	Χ^2^ = 1.6111 DF = 1 *p* = 0.2043	Χ^2^ = 0.0005 DF = 1 *p* = 0.9818
13	Χ^2^ = 4.0456 DF = 1 *p* = 0.0443	Χ^2^ = 0.0129 DF = 1 *p* = 0.9095	Χ^2^ = 3.5599 DF = 1 *p* = 0.0592
14	Χ^2^ = 0.2132 DF = 1 *p* = 0.6443	Χ^2^ = 0.6477 DF = 1 *p* = 0.4209	Χ^2^ = 1.5545 DF = 1 *p* = 0.2125
15	Χ^2^ = 0.0011 DF = 1 *p* = 0.974	Χ^2^ = 0.0702 DF = 1 *p* = 0.791	Χ^2^ = 0.0618 DF = 1 *p* = 0.8037
16	Χ^2^ = 0.3413 DF = 1 *p* = 0.5591	Χ^2^ = 2.7816 DF = 1 *p* = 0.0954	Χ^2^ = 0.4865 DF = 1 *p* = 0.4855
17	Χ^2^ = 3.7408 DF = 1 *p* = 0.0531	Χ^2^ = 0.1645 DF = 1 *p* = 0.6851	Χ^2^ = 4.1462 DF = 1 *p* = 0.0417
18	Χ^2^ = 0.5147 DF = 1 *p* = 0.4731	Χ^2^ = 0.4565 DF = 1 *p* = 0.4993	Χ^2^ = 0.007 DF = 1 *p* = 0.9334
19	-	-	-
20	Χ^2^ = 0.0303 DF = 1 *p* = 0.8618	Χ^2^ = 0.0914 DF = 1 *p* = 0.7624	Χ^2^ = 0.2192 DF = 1 *p* = 0.6397
21	Χ^2^ = 0.3264 DF = 1 *p* = 0.5678	Χ^2^ = 0.8467 DF = 1 *p* =0.3575	Χ^2^ = 0.2162 DF = 1 *p* = 0.6419
22	-	Χ^2^ = 1.6111 DF = 1 *p* = 0.2043	Χ^2^ = 2.0556 DF = 1 *p* = 0.1517
23	Χ^2^ = 1.2759 DF = 1 *p* = 0.2587	Χ^2^ = 0.6207 DF = 1 *p* = 0.4308	-

**Table 4:** Statistical comparison of serum BPA levels between study groups. Due to non-normal distribution (Shapiro–Wilk test), group comparisons were performed using non-parametric Mann–Whitney U tests. Ns—no significance.

BPA	Mean [pg/mL]	95% CI of Diff.	Significance	*p*-Value
CTRL vs. breast cancer	28,191.2 vs. 49,136	(−79,375 to 37,485)	Ns	0.877
CTRL vs. reproductive system cancer	28,191.2 vs. 95,883.4	(−132,721 to −2664)	Yes	0.045
Breast cancer vs. reproductive system cancer	49,136 vs. 95,883.4	(−108,081 to 14,586)	Ns	0.0884

## Data Availability

The original contributions presented in the study are included in the article. Further inquiries can be directed to the corresponding authors.

## Abbreviations

The following abbreviations are used in this manuscript:

BPA	bisphenol A
EDC	endocrine-disrupting chemical
ERR	estrogen-related receptor
PC	polycarbonate
PPAR	peroxisome proliferator-activated receptor
